# Associations of a Short Sleep Duration, Insufficient Sleep, and Insomnia with Self-Rated Health among Nurses

**DOI:** 10.1371/journal.pone.0126844

**Published:** 2015-05-11

**Authors:** Aline Silva-Costa, Rosane Härter Griep, Lúcia Rotenberg

**Affiliations:** 1 National School of Public Health, Oswaldo Cruz Foundation—ENSP/FIOCRUZ, Rio de Janeiro, Brazil; 2 Laboratory of Health and Environment Education, Oswaldo Cruz Institute—Fiocruz, Rio de Janeiro, Brazil; Centre for Chronobiology, SWITZERLAND

## Abstract

Epidemiological evidence suggests that sleep duration and poor sleep are associated with mortality, as well as with a wide range of negative health outcomes. However, few studies have examined the association between sleep and self-rated health, particularly through the combination of sleep complaints. The objective of this study was to examine whether self-rated health is associated with sleep complaints, considering the combination of sleep duration, insomnia, and sleep sufficiency. This cross-sectional study was performed in the 18 largest public hospitals in the city of Rio de Janeiro, Brazil. A total of 2518 female nurses answered a self-filled multidimensional questionnaire. The adjusted odds ratios and 95% confidence intervals (CIs) estimated the chance of poor self-rated health in the presence of different combinations of sleep duration and quality. Compared with women who reported adequate sleep duration with no sleep quality complaints (reference group), the odds ratios (95% CI) for poor self-rated health were 1.79 (1.27–2.24) for those who reported only insufficient sleep, 1.85 (0.94–3.66) for only a short sleep duration, and 3.12 (1.94–5.01) for only insomnia. Compared with those who expressed all three complaints (short sleep duration, insomnia, and insufficient sleep), the odds ratio for poor self-rated health was 4.49 (3.25–6.22). Differences in the magnitude of the associations were observed, depending on the combination of sleep complaints. Because self-rated health is a consistent predictor of morbidity, these results reinforce the increasing awareness of the role of sleep in health and disease. Our findings contribute to the recognition of sleep as a public health matter that deserves to be better understood and addressed by policymakers.

## Introduction

Sleep is increasingly recognized as a public health matter because sleep complaints affects millions of people [1]. The cumulative effects of sleep loss and sleep disorders need to be better understood and addressed [2,3]. The link between sleep and morbidity has been studied under the field of sleep medicine, which recognizes the importance of clinical characteristics of sleep [4].

Leproult and Van Cauter showed that sleep restriction results in metabolic and endocrine disorders, such as increased levels of nocturnal cortisol and ghrelin, and decreased leptin levels. These hormonal alterations might explain the effect of sleep duration on obesity epidemics [5]. The association of insomnia and the presence of glucose metabolism disorders and high blood pressure could serve as a potential biological explanation for the increase in cardiovascular risks observed in the presence of sleep debts and insomnia [6]. This link cannot be described as a linear cascade; rather, it is a complex and multifactorial process involving a neuro-endocrine-metabolic network [7]. Several studies have shown that sleep deprivation increases sympathetic nervous system activity, leading to increased blood pressure and heart rate [8,9]. Suarez et al. suggested that increased proinflammatory cytokine levels may also be involved in this matter [10]. Inadequate sleep may increase the cardiovascular risk in apparently healthy individuals because of activation of inflammatory processes, which could help explain the association between sleep complaints and cardiovascular morbidity [11].

Epidemiological evidence suggests that sleep duration and poor quality of sleep are associated with mortality [12], as well as with a wide range of negative health outcomes, including increased risks of hypertension, diabetes, obesity, depression, heart attack, and stroke [13–15]. A study on five European countries showed that night waking is associated with hypertension, cardiovascular diseases, diabetes, and high caffeine consumption [16]. Nagai et al. found evidence of an association between short sleep duration and diabetes mellitus, hypertension, and coronary heart disease [17]. A systematic review and meta-analysis of the longitudinal associations between sleep duration and all-cause mortality showed that a short duration of sleep is associated with greater risk of death (relative risk: 1.12; 95% confidence interval [CI]: 1.06–1.18), with no evidence of publication bias [18]. Grandner et al. highlighted the interaction between sleep duration and quality, which can increase the cardiometabolic risk [2]. This evidence indicates the need for considering not only the duration, but also aspects related to the quality of sleep, such as insomnia and insufficient sleep (fewer hours slept than what one believes to be sufficient to feel recovered).

Health can be assessed by self-rated health (SRH), which is considered a strong predictor of morbidity [19,20] and mortality [21–23]. Despite the consistent association between SRH and specific health outcomes, SRH has only been used in a few studies on sleep showing associations of poor self-rated health with insufficient rest/sleep [24], a short sleep duration [25,26], and sleep disturbance [27].

Therefore, the present study aimed to investigate the relationship between sleep and health considering three aspects of sleep that are potentially harmful to health: a short sleep duration, insomnia, and insufficient sleep. Health was assessed by SRH. We hypothesized that individuals who report a short sleep duration combined with insomnia and insufficient sleep are at a greater risk of reporting poor SRH than those who do not report such sleep problems.

## Methods

### Data collection

This cross-sectional study was performed in the 18 largest public hospitals in the city of Rio de Janeiro, Brazil. Data collection occurred from March 2010 to November 2011. Data were collected during work hours at the hospitals, through a comprehensive self-reported questionnaire that provided detailed information about the nursing job and health, as well as socioeconomic conditions [28].

### Ethical considerations

The study was briefly explained to participants who were informed that involvement was completely voluntary and that they could withdraw at any point in time with no negative consequences. All participants signed consent forms. The protocol was submitted and accepted by the Oswaldo Cruz Foundation (Fiocruz) Ethics Research Committee.

### Study population

The eligible study group comprised registered nurses providing assistance to patients at the hospitals. The nurses were invited to participate through a face-to-face approach by a team of interviewers [28]. Only female workers were included in analyses (n = 2818) because of gender differences in sleep complaints [29]. Responses were missing or invalid for 10.7% of the sample. The final sample included 2518 participants.

### Variables definition

#### 1. Sleep variables

- Sleep duration

The self-report questionnaire included the question “How many hours of sleep do you get in a usual night’s sleep?” Participants were classified according to sleep duration into two groups: the short sleep duration group (≤ 6 h) and adequate sleep duration group (7–9 h) [30,31].

- Insomnia

The following questions were asked: “In relation to your sleep during the night, at home, during the last 4 weeks, how often did you have difficulty in falling asleep?”, “…wake up and have difficulty going to sleep again?”, and “…wake up before the desired time and not manage to sleep again?” Response alternatives were never/rarely/sometimes (no complaint) and almost always/always (complaint). Respondents who reported any of the three described complaints were assigned to the insomniacs group [32,33].

- Sleep sufficiency

Evaluation of insufficient sleep was based on the following questions: “How many hours of sleep do you get in a usual night’s sleep?” and “On average, how many hours of sleep do you need each night to feel recovered?” A difference of 1 h or more was considered to indicate insufficient sleep [34]. Participants were classified into the sufficient sleep group (those who sleep enough to feel recovered) or insufficient sleep group (those who sleep less than they would like to feel recovered).

The exposure variable considered the combination of sleep duration, insomnia, and sleep sufficiency, comprising eight groups (Table 1). The first four groups included workers who reported adequate sleep duration, and they were characterized as (i) those who did not report any sleep complaints (i.e., those who reported an adequate sleep duration, sufficient sleep, and absence of insomnia), (ii) those who reported only insufficient sleep, (iii) those who reported only insomnia, and (iv) those who reported insufficient sleep and insomnia. The other groups were formed by those who reported a short amount of sleep, including (v) those who reported only short sleep duration, (vi) those who reported short sleep and insomnia, (vii) those who reported short and insufficient sleep, and (viii) those who reported short and insufficient sleep, and insomnia (Table 1).

**Table 1:** Exposure categories from the combination of three sleep complains.

Groups	Sleep	Duration	Insomnia		Sleep	Sufficiency	CATEGORIES OF SLEEP COMPLAINTS
	Adequate	Short	Non-insomniacs	Insomniacs	Sufficient	Insufficient	
1	X		X		X		Reference group (n = 500)
2	X		X			X	Only insufficient sleep (n = 464)
3	X			X	X		Only insomnia (n = 127)
4	X			X		X	Insufficient sleep and insomnia (n = 202)
5		X	X		X		Only short sleep (n = 65)
6		X		X	X		Short sleep and insomnia (n = 61)
7		X	X			X	Short and insufficient sleep (n = 583)
8		X		X		X	Short and insufficient sleep, and insomnia (n = 516)

#### 2. Self-rated health

SRH was evaluated by asking, “In general, compared with people of your age, would you say that your health is (…)”, with the following response categories: very good, good, fair, poor, and very poor. Among the five levels, “very good” and “good” were defined as the “healthy group” (reference group), while the remaining levels were defined as the “poor SRH group” [35].

### Statistical analysis

Descriptive statistics were used to describe exposure categories in relation to sociodemographic aspects, variables related to work, lifestyle, and health (physical exercise, smoking, age, marital status, income, professional work hours, shift work, alcohol consumption, caffeine consumption, sleep satisfaction, and body mass index [BMI]). Categorical variables are expressed as percentages and continuous variables are expressed as mean (standard deviation—SD). The chi-square test and analysis of variance were used to compare groups (significance level, *p* < 0.05). Variables showing at least minimal association (*p* < 0.10) were selected for inclusion in multiple models.

Logistic regression analyses were performed to test the association between the exposure categories and SRH. The first analysis included all exposure groups, thus considering each complaint separately, as well as a combination of complaints. In this analysis, workers who did not mention any complaints (group 1) were investigated as the reference group. The second analysis only included groups with a short sleep duration (groups 5 to 8), and aimed to determine whether insufficient sleep and insomnia would potentiate the association between short sleep and poor SRH. The group of those who reported only a short sleep duration (group 5) was considered as the reference group.

Results are presented as odds ratios and 95% CIs. All statistical analyses were performed using SPSS software (version 18.0; SPSS Inc., Chicago, IL, USA).

## Results

The mean age of participants was 39.9 (SD = 9.8) years (range, 22–68 years). Low income (<495 USD) was reported by 13.7% of the group and high income (>990 USD) was reported by 42.8%. Insufficient sleep was the most frequent complaint (70.1%), the prevalence of insomnia was 36%, and 48.6% of the women reported a short sleep duration. A total of 34.4% of women reported poor SRH.

Characteristics of the participants according to the combination of sleep duration, insomnia, and insufficient sleep are shown in Tables 2 and 3. Those with all three sleep complaints reported a higher percentage of poor SRH (51.9%) than did those who did not mention any complaints (18.9%), (p < 0.001). The prevalence of poor SRH was higher in those who reported only complaints of insomnia (40.2%) than in those who reported only a short sleep (30.8%) and only insufficient sleep (28.2%). High proportions of obesity and being married, and a low proportion of individuals who practiced physical activity were found in women who reported the combination of insomnia and insufficient sleep.

**Table 2:** Sociodemographic, occupational, and health characteristics of participants according to the combination of insomnia and insufficient sleep.

	Sleep 7–9 hours				
	Adequate sleep duration (n = 500)	Only insufficient sleep (n = 464)	Only insomnia (n = 127)	Insomnia and insufficient sleep (n = 202)	p value
**Age** (years − mean;SD)	40.5 (10.2)	38.3 (9.7)	39.8 (9.6)	39.4 (9.2)	0.004
**Income** (USD; %)					
< 495	12.5	12.9	17.2	13.8	0.836
495–990	44.8	41.8	42.2	42.9	
> 990	42.6	45.3	40.5	43.4	
**Marital status** (% married)	50.8	58.3	58.7	60.4	0.040
**Weekly work hours** (mean;SD)	53.7 (21.4)	53.9 (18.8)	52.4 (23.6)	54.2 (21.1)	0.882
**Work schedule** (%)					
Day workers	30.2	43.6	24.8	41.7	< 0.001
Night workers	69.8	56.4	75.2	58.3	
**Smoking** (%)					
Never smoked	78.1	80.5	76.1	76.2	0.803
Ex-smoker	14.8	13.2	14.4	16.3	
Current smoker	7.1	6.3	9.5	7.4	
**Alcohol consumption** (%)	56.8	62.7	56.1	66.9	0.075
**Physical activity** (% yes)	37.7	32.1	31.7	26.6	0.024
**Coffee consumption** (%)					
Low	24.7	24.5	14.6	19.9	0.017
Medium	59.8	59.7	57.7	60.7	
High	15.5	15.8	27.6	19.4	
**Sleep satisfaction** (%)					
Satisfied	80.8	64.9	53.2	38.3	< 0.001
Unsatisfied	19.2	35.1	46.8	61.7	
**BMI** (Kg/m^2^ − %)					
Eutrophyc	54.1	55.6	52.1	41.3	0.047
Overweight	28.7	25.9	29.3	34.6	
Obesity	17.2	18.5	18.6	24.1	
**Self-rated health** (%)					
Good	81.8	71.8	59.8	54.1	< 0.001
Poor	18.2	28.2	40.2	45.9	

**Table 3:** Sociodemographic, occupational, and health characteristics of participants according to the combination of short sleep, insufficient sleep, and insomnia.

	Sleep 7–9 hours	Short sleep duration (< = 6 hours)				
	Adequate sleep duration (n = 500)	Only short sleep duration (n = 65)	Short and Insufficient sleep (n = 583)	Short sleep and insomnia (n = 61)	Short and insufficient sleep and insomnia (n = 516)	p value
**Age** (years − mean;SD)	40.6 (10.2)	45.7 (9.4)	38.9 (9.5)	47.1 (9.6)	40.6 (9.9)	< 0.001
**Income** (USD; %)						
< 495	12.5	8.5	12.3	17.6	16.4	0.068
495–990	44.8	39.1	42.2	38.6	47.2	
> 990	42.6	52.4	45.5	43.9	36.4	
**Marital status** (% married)	50.8	51.6	54.8	49.9	60.2	0.039
**Weekly work hours** (mean;SD)	53.7 (21.4)	55.5 (21.7)	56.7 (20.1)	57.6 (24.1)	55.2 (21.1)	0.183
**Work schedule** (%)						
Day workers	30.2	37.5	46.9	37.3	47.2	< 0.001
Night workers	69.8	62.5	53.1	62.7	52.8	
**Smoking** (%)						
Never smoked	78.1	69.2	77.5	63.3	72.7	0.026
Ex-smoker	14.9	23.1	14.2	23.4	15.2	
Current smoker	7.0	7.7	8.4	13.3	12.1	
**Alcohol consumption** (%)	57.8	62.5	62.9	63.9	59.8	0.472
**Physical activity** (% yes)	37.9	34.4	30.2	30.1	22.5	< 0.001
**Coffee consumption** (%)						
Low	24.7	15.6	20.6	30.5	18.6	0.001
Medium	59.8	67.2	60.7	44.1	55.7	
High	15.5	17.2	18.7	25.4	25.7	
**Sleep satisfaction** (%)						
Satisfied	80.8	78.5	42.9	45.9	17.8	< 0.001
Unsatisfied	19.2	21.5	57.1	54.1	82.2	
**BMI** (Kg/m^2^ − %)						
Eutrophyc	54.1	35.5	49.8	35.7	47.8	0.005
Overweight	28.7	46.8	31.1	41.1	28.4	
Obesity	17.2	17.7	19.1	23.2	23.8	
**Self-rated health** (%)						
Good	81.8	69.2	66.6	73.8	48.1	< 0.001
Poor	18.2	30.8	33.4	26.2	51.9	

Table 4 shows the odds ratios and 95% CIs estimating the chance of poor SRH associated with different combinations of sleep duration and quality, adjusted for age, income, marital status, smoking status, alcohol consumption, physical activity, BMI, andwork schedule. Compared with women who reported an adequate sleep duration (reference group), the odds ratios for poor SRH were 1.79 (1.27–2.54) for those who reported only insufficient sleep, 1.85 (0.94–3.66) for only a short sleep duration, and 3.12 (1.94–5.01) for only insomnia. A significant interaction (*p* = 0.002) was observed between insomnia and sleep duration. In relation to the combined complaints, the odds ratios for poor SRH were 2.20 (1.59−3.04) for short sleep combined with insufficient sleep, 3.29 (2.20–4.93) for insufficient sleep combined with insomnia, and 4.49 (3.25–6.22) for all sleep problems together (short sleep duration, insomnia, and insufficient sleep) (Table 4).

**Table 4:** Odds ratios and 95% confidence intervals for poor self-rated health according to sleep complaints.

		Poor Self-rated Health		
		Model 1OR (95% CI)	Model 2OR (95% CI)	Model 3OR (95% CI)
**Adequate sleep duration**	No complaint	1	1	1
	Only insufficient sleep^†^	1.81 (1.30;2.51)	1.76 (1.26;2.45)	1.79 (1.27;2.54)
	Only insomnia^†^	3.32 (2.11;5.22)	3.24 (2.06;5.11)	3.12 (1.94;5.01)
	Insufficient sleep and insomnia^†^	3.65 (2.48;5.36)	3.59 (2.44;5.29)	3.29 (2.20;4.93)
**Short sleep duration**	Only short sleep^ǂ^	1.72 (0.89;3.34)	1.82 (0.94;3.54)	1.85 (0.94;3.66)
	Short and insufficient sleep^ǂ^	2.37 (1.74;3.23)	2.33 (1.71;3.18)	2.20 (1.59;3.04)
	Short sleep and insomnia^ǂ^	1.20 (0.57;2.51)	1.27 (0.60;2.66)	1.12 (0.52;2.44)
	Short and insufficient sleep, and insomnia^ǂ^	5.02 (3.69;6.85)	4.96 (3.64;6.77)	4.49 (3.25–6.22)

Model 1: unadjusted association; Model 2: adjusted for age, income, marital status; Model 3: adjusted for model 2 + physical activity, alcohol consumption^†^, coffee consumption^ǂ^, smoking habits, BMI and work schedule.

Table 5 shows results concerning women who reported a short sleep duration. There was a greater chance of reporting poor SRH among those who reported all of the complaints (OR = 2.40) compared with those who reported only a short sleep duration.

**Table 5:** Odds ratios and 95% confidence intervals for poor self-rated health according to sleep complaints in short sleepers.

	Poor Self-rated Health		
	Model 1OR (95% CI)	Model 2OR (95% CI)	Model 3OR (95% CI)
Only short sleep	1	1	1
Short and insufficient sleep	1.37 (0.72;2.60)	1.29 (0.68;2.47)	1.16 (0.60;2.25)
Short sleep and insomnia	0.70 (0.28;1.76)	0.70 (0.28;1.77)	0.62 (0.24;1.63)
Short and insufficient sleep, and insomnia	2.91 (1.54;5.52)	2.75 (1.44;5.26)	2.40 (1.24;4.65)

Model 1: unadjusted association; Model 2: adjusted for age, income, marital status; Model 3: adjusted for model 2 + smoking habits, physical activity, BMI, coffee consumption and work schedule.

## Discussion

The current study investigated the combination of quality and duration of sleep in relation to SRH. We found that all sleep complaints were separately associated with poor SRH. Differences in the magnitude of the odds ratios for each unique complaint showed that insomnia was the most strongly associated with poor SRH, followed by a short sleep duration, and insufficient sleep. We highlight the main contribution of this study, which is the combined analysis of the quality and duration of sleep in relation to the SRH. Actually, even among individuals who reported an adequate sleep duration, the combination of insufficient sleep and insomnia was associated with a high chance of poor SRH (odds ratio = 3.29); inclusion of a short sleep duration led to an increase in the adjusted odds ratio to 4.49, potentiating the estimates for poor SRH. These results suggest that sleep duration may interact with sleep quality, intensifying the risk of diseases [2].

Other studies have reported an association between a short sleep duration and poor SRH. Steptoe et al. observed that the adjusted odds ratio (OR) for poor SRH was 1.99 for people sleeping less than 6 h and 1.56 for those sleeping 6 to 7 h compared with 7–8 h of daily sleep [36]. In a large multiethnic sample of US adults, multivariate odds ratios of fair/poor SRH among women were 2.52 and 1.74 for a sleep duration ≤ 5 h and 6 h, respectively, compared with 7 h of daily sleep [25]. A cross-sectional study of middle-aged and older Australian adults showed that < 6 h of sleep and 6 h of sleep were associated with poor SRH (odds ratios were 1.49 and 1.28, respectively) [37].

Sleep quality has also been shown to be associated with SRH. A higher quality of sleep is associated with a very good (OR = 2.65) and good (OR = 2.88) SRH [38]. The odds ratio for poor SRH was shown to be 2.6 in those with poor sleep quality [39]. Geiger et al. [24] showed a dose-response association of insufficient sleep/rest and SRH. They found that the odds ratio for poor SHR was 1.45 for 7–13 days of insufficient sleep/rest, and odds ratios were 2.12, 2.32, and 2.71 for 14–20 days, 15–29 days, and 30 days of insufficient sleep/rest, respectively. The significant association of insufficient sleep/rest and SRH persisted within stratified subgroups of gender, age, race-ethnicity, and BMI [24]. Data obtained by Grandner et al. showed that better general health was associated with sleep-related complaints, regardless of age and other factors [27].

The underlying mechanism linking poor SRH to a short sleep duration and quality is not fully understood. SRH is a broad concept and likely reflects various combinations of many potential pathways. With regard to sleep problems and negative health outcomes, several studies have shown that sleep deprivation increases sympathetic nervous system activity, leading to increased blood pressure and heart rate [8,9]. Sleep restriction results in metabolic and endocrine disorders [5]. According to Meier-Ewert et al., sleep loss may increase the cardiovascular risk in apparently healthy individuals because of activation of inflammatory processes, which could contribute to the association between sleep complaints and cardiovascular morbidity [11]. The association between poor SRH and mortality may partially be mediated by insufficient sleep [24]. Additionally, poor SRH may be a mediator of the associations among sleep duration, cardiovascular disease, and mortality [39].

In our study, differences in the magnitude of the associations were identified because of the method used to subdivide the sample into eight groups. This allowed us to analyze the relationship between sleep quality and SRH, even among those with an adequate sleep duration. Among those with an adequate sleep duration, many women reported complaints of insufficient sleep, insomnia, or both (Table 1). The methodological procedure that we used was beneficial compared with methods that were usually adopted in the literature, in which sleep duration studies describe the reference category without considering sleep quality, which can lead to an underestimation of associations. Heterogeneity in the quality of sleep among people with an adequate sleep duration indicates the need to include aspects of sleep quality in studies on the relationship between sleep and health.

The current study assumed that sleep problems may lead to poor SRH through, among other factors, the influence of sleep complaints on daytime fatigue or on a decrease of restorative sleep function [36]. Poor health may also lead to a short sleep duration and poor quality of sleep. Therefore, individuals with poor health are more likely to have sleep complaints and are less able to estimate their sleep duration [25]. Accordingly, causal conclusions cannot be drawn from this cross-sectional study. Another limitation of this study is related to sleep-disordered breathing, which is the second most common sleep disorder, and a potential confounder that was not investigated. Additionally, sleep duration was based on self-reporting, which may have involved errors, such as reflecting time in bed instead of real sleep duration [40]. Although measuring objective sleep duration is more accurate, this procedure is usually not feasible in large epidemiological samples. Sleep studies based on polysomnography, the ‘‘gold standard” method, or wrist activity monitoring (actigraphy) [41] for recording sleep could be useful for verifying our findings. Notably, generalization of our findings should be made with caution because nurses are a specialized group of workers who are submitted to long professional work hours and shift work, and these aspects can influence health.

Finally, because SRH is a consistent predictor of morbidity and mortality, our results reinforce the increasing awareness of the role of sleep in health and disease [30]. Actually, the clinical relevance of sleep characteristics is currently recognized by the scientific community through maturation of the field of sleep medicine [42]. In summary, our results contribute to recognition of sleep as a public health problem that deserves to be better understood and addressed by policymakers.
